# Proximity‐Unlocked Luminescence by Sequential Enzymatic Reactions from Antibody and Antibody/Aptamer (PULSERAA): A Platform for Detection and Visualization of Virus‐Containing Spots

**DOI:** 10.1002/advs.202403871

**Published:** 2024-09-24

**Authors:** Daimei Miura, Wakana Hayashi, Kensuke Hirano, Ikkei Sasaki, Kaori Tsukakoshi, Hidehumi Kakizoe, Satomi Asai, Christopher J. Vavricka, Hitoshi Takemae, Tetsuya Mizutani, Wakako Tsugawa, Koji Sode, Kazunori Ikebukuro, Ryutaro Asano

**Affiliations:** ^1^ Department of Biotechnology and Life Science Graduate School of Engineering Tokyo University of Agriculture and Technology 2‐24‐16 Naka‐cho Koganei Tokyo 184–8588 Japan; ^2^ Institute of Global Innovation Research Tokyo University of Agriculture and Technology 3‐8‐1 Harumi‐cho Fuchu Tokyo 183–8538 Japan; ^3^ Department of Industrial Technology and Innovation Graduate School of Engineering Tokyo University of Agriculture and Technology 2‐24‐16 Naka‐cho Koganei Tokyo 184–8588 Japan; ^4^ Department of Laboratory Medicine Tokai University School of Medicine 143 Shimokasuya Isehara Kanagawa 259–1193 Japan; ^5^ Division of Infection Control Tokai University Hospital 143 Shimokasuya Isehara Kanagawa 259–1193 Japan; ^6^ Center for Infectious Disease Epidemiology and Prevention Research Faculty of Agriculture Tokyo University of Agriculture and Technology 3‐5‐8 Saiwai‐cho Fuchu Tokyo 183–8509 Japan; ^7^ Cooperative Division of Veterinary Sciences Graduate School of Agriculture Tokyo University of Agriculture and Technology 3‐5‐8 Saiwai‐cho Fuchu Tokyo 183–8509 Japan; ^8^ Joint Department of Biomedical Engineering University of North Carolina at Chapel Hill and North Carolina State University Chapel Hill NC 27599 USA

**Keywords:** antibody‐enzyme complex, bispecific aptamer, homogeneous immunosensor, sequential enzymatic reactions, virus detection and visualization

## Abstract

The SARS‐CoV‐2 pandemic has challenged more scientists to detect viruses and to visualize virus‐containing spots for diagnosis and infection control; however, detection principles of commercially available technologies are not optimal for visualization. Here, a convenient and universal homogeneous detection platform named proximity‐unlocked luminescence by sequential enzymatic reactions from antibody and antibody/aptamer (PULSERAA) is developed. This is designed so that the signal appears only when the donor and acceptor are in proximity on the viral surface. PULSERAA specifically detected in the range of 25–500 digital copies/mL of inactivated SARS‐CoV‐2 after simply mixing reagents; it is elucidated that the accumulation of chemical species in a limited space of the viral surface contributed to such high sensitivity. PULSERAA was quickly adapated to detect another virus variant, inactivated influenza A virus, and infectious SARS‐CoV‐2 in a clinical sample. Furthermore, on‐site (direct, rapid, and portable) visualization of the inactivated SARS‐CoV‐2‐containing spots by a conventional smartphone camera was achieved, demonstrating that PULSERAA can be a practical tool for preventing the next pandemic in the future.

## Graphical abstract

A homogeneous immunoassay named proximity‐unlocked luminescence by sequential enzymatic reactions from antibody and antibody/aptamer (PULSERAA) is developed, which enabled not only rapid virus detection in 15 min with high sensitivity and specificity but also the on‐site visualization of the virus‐containing spots on a surface through a smartphone camera after spraying reagents.

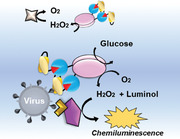

## 1 Introduction

Since the severe acute respiratory syndrome coronavirus 2 (SARS‐CoV‐2) pandemic began in 2019, easy detection and recognition of trace amounts of toxic pathogens such as viruses are increasingly being targeted not only for diagnosis, but also for prevention of infections. Accordingly, visualization of pathogen‐containing spots with the naked eye or a camera with specific filters is becoming a critical aspect of modern healthcare, particularly for infection control in the next pandemic. However, the existing methods require bound/free (B/F) separation procedures, such as physical washing in enzyme‐linked immunosorbent assays (ELISA)^[^ ^1^, ^2^ ^]^ and magnetic separation in immunomagnetic assays,^[^ ^3^ ^]^ rendering them time‐consuming and inconvenient. These procedures often require well‐trained technicians and relatively large equipment such as plate readers and spectrometers. Lateral flow immunoassays have become very mature system for early detection and enable performance by untrained users owing to easy B/F separation.^[^ ^4^ ^]^ However, they also require sample collections, which is another major obstacle for direct visualization of virus‐containing spots on a surface. The forensic luminol test for blood^[^ ^5^ ^]^ is based on detection of luminol chemiluminescence in the presence of heme supplied by hemoglobin in the bloodstain and added hydrogen peroxide, which enables the visualization of blood‐containing spots. This involves simply spraying a solution from a conventional dispenser, but convenient visualization‐detection systems for other biomarkers such as viruses are lacking.

Homogeneous assays do not require B/F separation.^[^ ^6^ ^]^ Fluorescence resonance energy transfer (FRET)‐based immunoassays and the AlphaLISA® system, derived from the amplified luminescent proximity homogeneous assay (AlphaScreen®) technology,^[^ ^7^ ^]^ have received attention as a powerful tool for homogeneous assays.^[^ ^8^, ^9^ ^]^ Both techniques intrinsically rely on the proximity of two molecules, a donor and an acceptor, and the signal transfer between them, which enables a homogeneous assay, as signals generated from donors unbound to targets cannot reach distant acceptors. However, these assays are based on fluorescence detection, which requires relatively large and specialized equipment including the light source for excitation for signal measurement, hindering their application for convenient detection or visualization. In terms of the signal relay system, immunosensors based on sequential enzymatic reactions have already been reported using glucose oxidase (GOx) and horseradish peroxidase (HRP) or other combinations of enzymes and enzyme‐mimicking molecules.^[^ ^10^, ^11^, ^12^ ^]^ The sequential enzymatic reaction has an advantage in signal amplification for sensitive detection,^[^ ^11^, ^13^ ^]^ but it inherently requires B/F separation procedures to obtain the target‐specific signal, as well as conventional sandwich ELISA.

Combinations of molecular recognition elements with high binding affinities and specificities, and enzymes with high activities for signal transduction, are the most suitable methods for detecting targets with high accuracy and sensitivity. We recently developed a universal and convenient method for the preparation of antibody‐enzyme complexes (AEC) using the SpyCatcher (SC)/SpyTag (ST) system^[^ ^14^ ^]^; AECs were fabricated using small antibodies, the variable domain of the heavy chain of the heavy chain antibody (VHH) or single‐chain variable fragment (scFv) as the recognition elements, and glucose dehydrogenase (GDH) from *Aspergillus flavus* as the enzyme. An electrochemical detection method using these AECs and magnetic beads were established, as GDH is an oxidoreductase that can generate electrochemical signals upon enzymatic reactions,^[^ ^15^, ^16^, ^17^ ^]^ which enabled reduction of the number of washing steps for rapid and convenient detection. However, B/F separation using a magnet and sample collection are still required, and therefore these methods cannot be used for convenient visualization in daily healthcare practices.

In this study, we developed a convenient, universal, and homogeneous viral detection and visualization platform based on the sequential enzymatic reaction between oxidases and peroxidases on a viral surface, named proximity‐unlocked luminescence by sequential enzymatic reactions from antibody and antibody/aptamer (PULSERAA). Three variations of PULSERAA systems were constructed with different acceptor molecules and evaluated as homogeneous immunosensors for the detection and visualization of virus‐containing spots. PULSERAA could specifically detect inactivated SARS‐CoV‐2, and the system could be readily expanded to detect another SARS‐CoV‐2 variant and influenza A virus by replacing the recognition elements. In addition, we detected infectious SARS‐CoV‐2 from a clinical throat swab. Finally, we succeeded in visualizing the virus‐containing spots on a surface using a camera that was originally incorporated into a smartphone after spraying the PULSERAA reagent. These results demonstrate that PULSERAA is a novel platform that can be used for glucose‐fueled, rapid, convenient, and highly sensitive virus detection and visualization of infectious spots on a surface, which will enable early diagnosis and prevention of infections in the next pandemic.

## 2 Results and Discussion

### 2.1 Design of PULSERAA System

**Figure 1:**
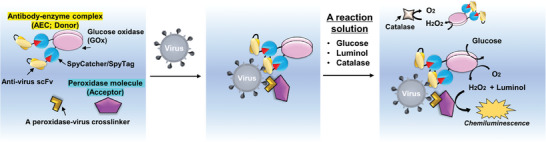
A schematic illustration of the PULSERAA system. The main components are the antibody‐enzyme complex (AEC) as the donor, the peroxidase molecule as the acceptor, and a peroxidase‐virus crosslinker. These components associate with each other in the presence of targets, such as viruses. After incubation, a reaction solution containing glucose, luminol, and catalase is added to initiate the first enzymatic reaction in the AEC. Then, the produced hydrogen peroxide is relayed to the peroxidase molecule, where a second enzymatic reaction occurs with the emission of luminol‐derived chemiluminescence. Catalase acts as a scavenger that removes hydrogen peroxide generated by unbound AEC, thereby decreasing the background signal.

Sensitive immunoassays such as ELISA are achieved by B/F separations, but they often take a long time and require cumbersome procedures. To eliminate the B/F separation steps and achieve sensitive detection, we focused on sequential enzymatic reactions that occur only in limited spaces. In other words, the migration of hydrogen peroxide generated from a donor to an acceptor in proximity within limited spaces, such as on virus surfaces, allows the emission of optical signals such as chemiluminescence, because the sequential enzymatic reactions can be induced locally, leading to development of a homogeneous assay and the first system to visualize virus‐containing spots. Therefore, we employed an oxidase‐based AEC as the donor and a peroxidase‐based element as the acceptor in the PULSERAA system (**Figure** **1**, left panel). Here, we used GOx from *Aspergillus niger* as the donor oxidase of the AEC because glucose is non‐toxic and highly stable, and GOx has been widely used as a promising molecule in glucometers worldwide due to its high oxidase activity.^[^ ^18^ ^]^ In terms of the acceptor with peroxidase activity, three molecules, myoglobin (Mb), hemin, and HRP were explored to construct the versatile detection system, where the difference in the molecule with the peroxidase activity determines the version of the PULSERAA system.

The general detection principle is as follows. The donor and acceptor come into proximity when they recognize the target virus (Figure 1, center panel). Then, a reaction solution containing glucose, luminol, and catalase is added; catalase acts as a scavenger to degrade hydrogen peroxide produced by AECs unbound to the virus. Thereafter, sequential enzymatic reactions occur only where the antigenic targets accumulate on the virus surface, where chemiluminescence increases in a virus concentration‐dependent manner. Accordingly, PULSERAA is a homogeneous immunoassay (Figure 1, right panel).

As for PULSERAA version 1.0, we used Mb as the acceptor. To crosslink Mb and the virus and to bring the AEC and Mb into proximity, we used a bispecific aptamer that can bind to Mb and the surface protein of the virus. After incubation of Mb, the bispecific aptamer, the AEC, and the virus, a detection solution is added, and chemiluminescence is measured subsequently. More detailed experimental information can be obtained in the Experimental Section.

### 2.2 Preparation of scFv‐SpyTag Targeting the Surface of SARS‐CoV‐2

**Figure 2:**
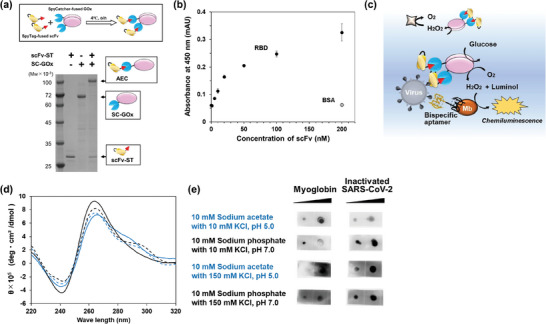
a) A schematic illustration of SpyCatcher/SpyTag reaction with SpyTag (ST)‐fused scFv and SpyCatcher (SC)‐fused glucose oxidase (GOx) and SDS‐PAGE analysis for confirming the AEC formation. As GOx is a non‐covalent homo‐dimeric protein, each protein migrates to the position corresponding to the molecular weight of the monomeric protein under reducing condition. b) Evaluation of the binding ability of anti‐RBD scFv‐ST using ELISA. Bovine serum albumin (BSA) was used as the negative control. Data are represented the mean ± S.D. (*n* = 3). c) A schematic illustration of PULSERAA version 1.0 when myoglobin (Mb) was used as the acceptor. d) Circular dichroism (CD) spectrum analysis of the bispecific aptamer (PEA3‐01–RBD‐46) and the effect of folding buffer conditions on its tertiary structure. The result obtained from the aptamer folded in a buffer of pH 5.0 is shown in blue and that at pH 7.0 in black. The lower concentration of K^+^ is shown as a solid line and the higher concentration as a dotted line. e) Analysis of the binding ability of the bispecific aptamer (PEA3‐01–RBD‐46) folded in each buffer. For the assay, 0.35 and 3.5 µg Mb, and 5 and 20 dC inactivated SARS‐CoV‐2 were used on nitrocellulose membranes.

The receptor‐binding domain (RBD) of SARS‐CoV‐2 was selected as the target for viral detection. Based on the reported amino acid sequence of the Fab format of an anti‐RBD antibody,^[^ ^19^ ^]^ ST‐fused scFv, denoted as anti‐RBD scFv‐ST, was designed by connecting both variable regions (VH‐VL order) with a flexible linker, and ST was fused to its C‐terminus as reported previously.^[^ ^14^ ^]^ As the yield of scFv‐ST from culture supernatant was low, the scFv‐ST was prepared via refolding processes and purified using gel filtration chromatography (Figure S1a, Supporting Information). Sodium dodecyl sulfate‐polyacrylamide gel electrophoresis (SDS‐PAGE) revealed that the highly purified scFv‐ST was successfully prepared with a high yield of 1.9 mg L^−1^ culture (**Figure** **2**a; left lane).

The binding ability of anti‐RBD scFv‐ST was evaluated using ELISA. As shown in Figure 2b, the absorbance at 450 nm increased in a concentration‐dependent manner, and the signal for bovine serum albumin (BSA) was the same as that of the background, indicating that the prepared anti‐RBD scFv‐ST had high affinity and specificity for the RBD of SARS‐CoV‐2.

### 2.3 SpyCatcher‐Fused GOx Preparation, AEC Fabrication, and Functional Analysis

SC was fused to the N‐ or C‐terminus of GOx derived from *A. niger*, and the fusion proteins were denoted as SC‐GOx and GOx‐SC, respectively. As the expression of both proteins was observed in the insoluble fraction, they were prepared via refolding processes with high purity (Figure 2a; center lane). The productivities and refolding efficiencies of SC‐GOx and GOx‐SC were 33 mg L^−1^ and 66%, and 36 mg L^−1^ and 72%, respectively.

Anti‐RBD scFv‐ST and SC‐fused GOx were mixed stoichiometrically and incubated overnight at 4 °C. The formation of AECs, prepared using the SC/ST system (Figure 2a, top figure), was evaluated using SDS‐PAGE analysis (Figure 2a, bottom figure). The result obtained using SC‐GOx is shown as representative data. Each band corresponding to anti‐RBD scFv‐ST and SC‐GOx almost disappeared after incubation, and a band corresponding to AEC was observed (Figure 2a; right lane), indicating that the AEC was fabricated by incubating at 4 °C.

The AEC bifunctionality was then evaluated. First, oxidase activity was analyzed (Figure S1b, Supporting Information; top figure), and the calculated kinetic parameters, *K*_M_ and *V*_max_, are summarized in **Table** **1**.

**Table 1:** Kinetic parameters of the oxidase activities of GOx, SpyCatcher (SC) fusion proteins, and antibody‐enzyme complexes (AECs) composed of GOx and anti‐RBD scFvs.

	*K*_M_ [mm]	*V*_max_ [U nmol^−1^]
GOx	33	3.6
SC–GOx	32	3.0
GOx‐SC	31	2.7
AEC (scFv‐GOx)	30	2.6
AEC (GOx‐scFv)	37	2.3

These parameters did not change much after the SC fusion, followed by AEC formation. Therefore, we conclude that the fusion of SC and the AEC formation does not greatly affect oxidase activity. Then, the influence of pH on oxidase activity was evaluated. The oxidase activities decreased in response to increase in pH, and almost 50% of activity was maintained at pH 8.0 compared to pH 6.5, while that of SC‐GOx was slightly higher than that of GOx‐SC (Figure S1b, Supporting Information; bottom figure). Our previous studies have shown that the AEC composed of GDH from *A. flavus* and small antibodies can be easily prepared using the SC/ST system,^[^ ^14^, ^15^, ^16^, ^17^ ^]^ and expanded the use for the fabrication of DNA‐enzyme complex, where GOx from *A. niger* was used as an enzyme.^[^ ^20^ ^]^ These AECs were prepared without any loss of enzymatic functions, which shows high compatibility with SC/ST system.

Next, the affinities of anti‐RBD scFv‐ST and AEC were analyzed using biolayer interferometry (BLI) by fitting of the data to the 1:1 Langmuir global model (**Table** **2** and Figure S1c, Supporting Information). The association rate constants (*k*_on_) of anti‐RBD scFv‐ST and the AEC were 4.2 × 10^4^ M^−1^s^−1^ and 3.1 × 10^4^ M^−1^s^−1^, respectively, suggesting that the association of each antibody with the RBD was not largely affected by the AEC formation. In contrast, the dissociation rate constants (*k*_off_) of anti‐RBD scFv‐ST and the AEC were 1.4 × 10^−3^ s^−1^ and 5.8 × 10^−4^ s^−1^, respectively, and the dissociation constants (*K*_D_) of anti‐RBD scFv‐ST and the AEC were 32 and 19 nm, respectively. This is a typical example of a bivalent effect,^[^ ^21^ ^]^ as GOx has a dimeric structure and two scFvs can be associated with the SC‐fused GOx.

**Table 2:** Kinetic parameters of the oxidase activities of GOx, SpyCatcher (SC) fusion proteins, and antibody‐enzyme complexes (AECs) composed of GOx and anti‐RBD scFvs.

	*k*_on_ [M^‐1^s^‐1^]	*k*_off_ [s^‐1^]	*K*_D_ [M]	Chi^2^
Anti‐RBD scFv‐ST	4.2 × 10^4^ ± 0.11 × 10^4^	1.4 × 10^−3^ ± 0.052 × 10^−3^	3.2 × 10^−8^ ± 0.15 × 10^−8^	0.23
AEC (scFv‐GOx)	3.1 × 10^4^ ± 0.12 × 10^4^	5.8 × 10^−4^ ± 0.52 × 10^−4^	1.9 × 10^−8^ ± 0.18 × 10^−8^	0.26

Taken together, the prepared AEC was bifunctional, retaining 87% of the oxidase activity even after the AEC formation, and showing enhanced binding ability to the RBD of SARS‐CoV‐2 due to the bivalent effect, which strongly indicates that it is an optimal molecule for detection of SARS‐CoV‐2.

### 2.4 Functional Evaluations of an Acceptor used for PULSERAA Version 1.0

We reported a Mb‐binding DNA aptamer, PEA3‐01^[^ ^22^ ^]^; hence, we designed PULSERAA version 1.0 by integrating a DNA aptamer and Mb as the acceptor (Figure 2c). Thereafter, we prepared a bispecific aptamer that binds to the RBD of SARS‐CoV‐2 and Mb, such that the AEC and Mb come into proximity on the viral surface in the presence of the virus. An RBD‐binding aptamer (RBD‐46) was obtained by truncating partial sequences of an aptamer, CoV2‐RBD‐4C,^[^ ^23^ ^]^ and we prepared bispecific aptamers (PEA3‐01–RBD‐46 and RBD‐46–PEA3‐01). All the aptamer sequences used in this study are summarized in Table S1 (Supporting Information). The bispecific aptamers were first folded in 10 mm potassium acetate buffer (pH 5.0) according to the previous report,^[^ ^22^ ^]^ and circular dichroism (CD) spectra were recorded to evaluate the tertiary structures of the bispecific aptamers. As shown in Figure S1d (Supporting Information), a positive peak at 280 nm and a negative peak at 240 nm were observed for both bispecific aptamers, indicating that they formed a mixed structure of parallel and antiparallel G‐quadruplex (G4) structures, or a hybrid (3 + 1) G4 structure.^[^ ^24^ ^]^ Then, the binding abilities to each target, Mb, and inactivated SARS‐CoV‐2 Wuhan strain (Munich/ChVir984/2020) were analyzed. The results showed that both bispecific aptamers bound to Mb and the inactivated SARS‐CoV‐2 (Figure S1e, Supporting Information), and that a higher binding signal was observed when using PEA3‐01–RBD‐46 at lower concentration of Mb; therefore, PEA3‐01–RBD‐46 was used for further experiments.

Next, we investigated suitable folding conditions for the bispecific aptamer because PEA3‐01–RBD‐46 formed heterogeneous structures. As a result, the bispecific aptamer folded in a buffer supplemented with 10 mm Na^+^ and 10 mm K^+^ (pH 7.0) showed clear negative and positive peaks near 240 and 260 nm, respectively, whereas bispecific aptamers folded under other conditions showed an additional positive peak near 290 nm (Figure 2d). The binding ability of the bispecific aptamer folded in the buffer containing 10 mm Na^+^ and 10 mm K^+^ (pH 7.0) showed the highest binding to lower concentration of Mb, as well as to the inactivated SARS‐CoV‐2 (Figure 2e). These results demonstrate that the bispecific aptamer folded in the presence of 10 mm Na^+^ and 10 mm K^+^ (pH 7.0) adopts a uniform parallel G4 structure, which contributes to its improved binding ability and the possibility of highly sensitive detection in PULSERAA version 1.0.

### 2.5 Homogeneous Detection of Inactivated SARS‐CoV‐2 using PULSERAA Version 1.0

The most important feature of PEA3‐01 is that the innate peroxidase activity of Mb is dramatically enhanced by up to 300‐fold upon aptamer binding.^[^ ^22^ ^]^ Hence, we investigated the mixing ratio of Mb and the bispecific aptamer in the presence of a constant concentration of hydrogen peroxide to maximize chemiluminescence emission. As shown in Figure S2a (Supporting Information), the addition of five times the amount of the bispecific aptamer to Mb exhibited the highest chemiluminescence intensity. Next, we investigated whether or not catalase improves the signal/background ratio by eliminating hydrogen peroxide produced from unbound AECs to enable detection without any washing procedures. As shown in Figure S2b (Supporting Information), a higher signal/background ratio was observed in the presence of catalase. In addition, the background signal in the presence of catalase was significantly decreased (Figure S2c, Supporting Information). These results show that catalase works as a scavenger to improve the performance of the PULSERAA system by decreasing background signals. In the PULSERAA system, 1.2 × 10^−2^ U of catalase and 1.3 × 10^−2^ U of the AEC were contained in the final solution in a well of a 96‐well plate, which means that the theoretical amount of hydrogen peroxide produced from the AEC should be equal to that consumed by the catalase, suggesting that peroxidase used as the acceptor in the PULSERAA system will compete with catalase to consume hydrogen peroxide. Considering the *K*_M_ values of catalase (66.7 mm),^[^ ^25^ ^]^ Mb (20 mm),^[^ ^26^ ^]^ and other peroxidase molecules, hemin (7.28 × 10^−2^ mm)^[^ ^27^ ^]^ and HRP (2.2 × 10^−3^ mm),^[^ ^28^ ^]^ the substrate affinities of the acceptors to hydrogen peroxide are higher than that of catalase. Therefore, the competing acceptor enzymes in proximity to the AEC on the viral surface can still react with hydrogen peroxide, characterizing the PULSERAA system as a homogeneous detection with high sensitivity by decreasing background signals.

**Figure 3:**
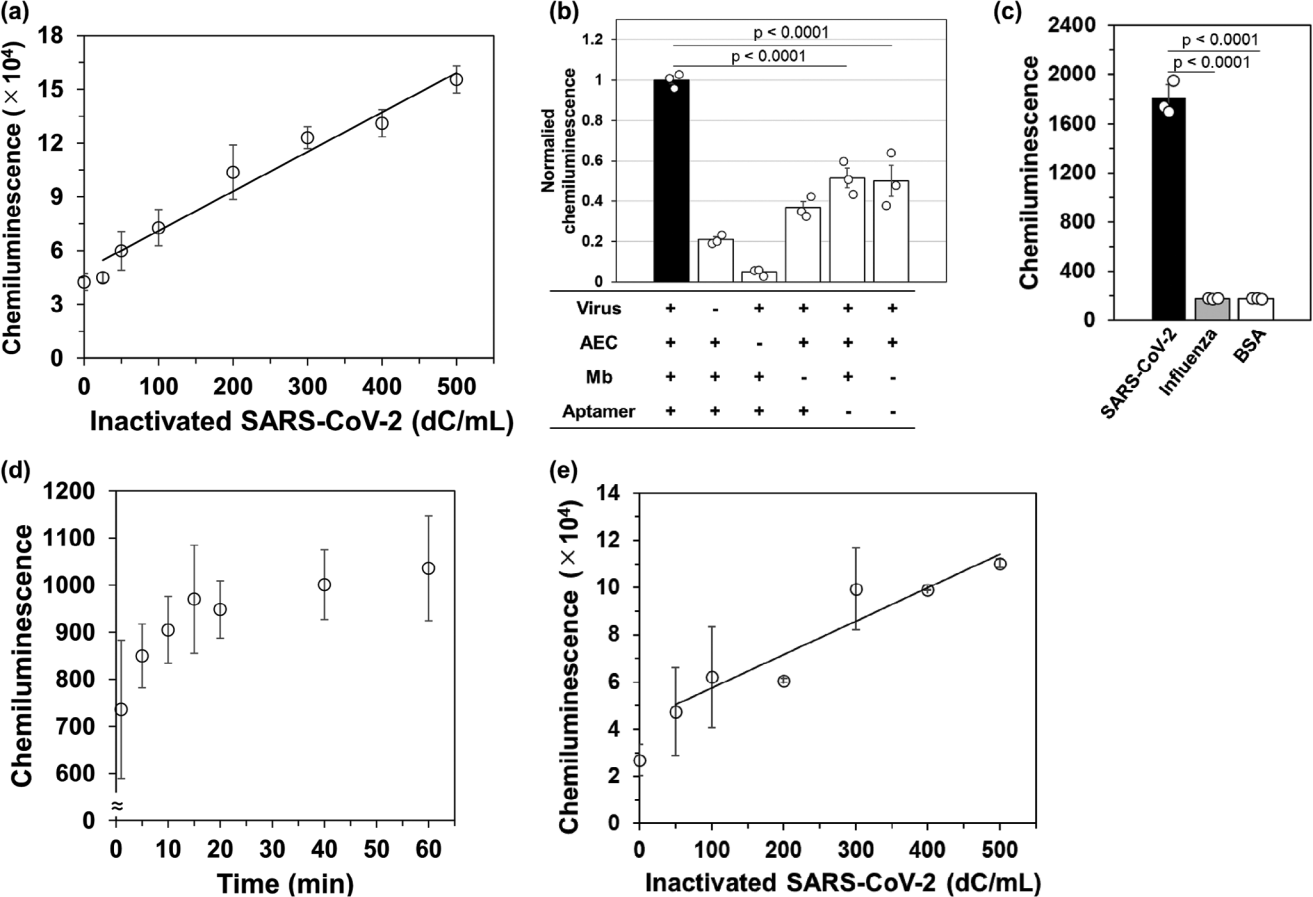
a) Detection of inactivated SARS‐CoV‐2 using PULSERAA version 1.0 using a commercially available chemiluminescence reagent supplemented with 200 mm glucose and 24 ng mL^−1^ catalase to be the final concentration. The data are represented as mean ± S.D. (*n* = 3; R^2^ = 0.984). b) Evaluation of the necessity of each component in the PULSERAA version 1.0 to check the proposed mechanism of hydrogen peroxide relay via sequential enzymatic reactions at the virus surface. Chemiluminescence values for incomplete conditions were normalized by assigning the chemiluminescence signal of the condition containing all components as 1. Normalized data are represented as mean ± S.E. (*n* = 3). c) Specificity of the detection using PULSERAA version 1.0. The data are represented as mean ± S.D. (*n* = 3). d) Investigation of the incubation time for the detection of inactivated SARS‐CoV‐2 using PULSERAA. Each component was mixed and incubated for 1–60 min, and chemiluminescence intensity was quantified immediately after the incubation. The data are shown as mean ± S.D. (*n* = 3). e) The detection of inactivated SARS‐CoV‐2 in 15 min incubation. The data are shown as mean ± S.D. (*n* = 3; R^2^ = 0.947).

Next, we performed the proof‐of‐concept detection of inactivated SARS‐CoV‐2 using PULSERAA version 1.0, using a custom detection solution. As shown in Figure S2d (Supporting Information), inactivated SARS‐CoV‐2‐dependent chemiluminescence increase was observed, and a linear range of 25–500 digital copies (dC)/mL was observed, where the unit of dC is suggested to be determined by digital PCR.^[^ ^29^ ^]^ However, the sensitivity that can be defined as the slope of the calibration curve was relatively low (1.0 dC mL^−1^). Therefore, we tested an optimized commercial chemiluminescence reagent to improve the detection of inactivated SARS‐CoV‐2. As a result, inactivated SARS‐CoV‐2 was successfully detected by a viral concentration‐dependent increase in chemiluminescence with the same linear range of 25–500 dC mL^−1^, but with improved sensitivity (220 dC mL^−1^) (**Figure** **3**a) where the limit of detection (3σ LOD) was calculated to be 31 dC mL^−1^. Here, σ is the standard deviation of the chemiluminescence derived from 0 dC mL^−1^ inactivated SARS‐CoV‐2. These results emphasize the importance of using an optimized detection reagent to achieve high sensitivity in our system. Then, we confirmed that the absence of any of the components resulted in significant decrease of chemiluminescence emission (Figure 3b). The absence of the AEC in the detection system resulted in a weak chemiluminescent signal (5.1%), indicating that GOx in the AEC is the only hydrogen peroxide supplier. Chemiluminescence significantly decreased without Mb (37%), without the bispecific aptamer (52%), and without both of Mb and the bispecific aptamer (50%). This indicates the importance of Mb and AEC in proximity to the virus, which contributes to signal separation in the homogeneous immunoassay, and that the use of PEA3‐01 slightly contribute to enhance the chemiluminescence. On the other hand, these chemiluminescence was relatively high (≈50%), suggesting that the solution containing inactivated SARS‐CoV‐2 has some component with peroxidase activity and that it binds to the viral surface non‐specifically. These results elucidate that the PULSERAA system is dependent on the two‐enzyme cascade reaction when the donor and acceptor are in proximity on the viral surface.

The specificity of the detection was also evaluated. As a result, a chemiluminescent signal was not observed when the inactivated influenza A virus (A/Taiwan/1/86) and BSA were incubated instead of the inactivated SARS‐CoV‐2 (Figure 3c). In addition, chemiluminescence increased in a time‐dependent manner and reached a plateau after 15 min of incubation (Figure 3d). Therefore, we performed the detection in 15 min, and a linear range of 50–500 dC mL^−1^ and the LOD of 23 dC mL^−1^ was observed (Figure 3e), suggesting that the detection time can be shortened within 15 min. To obtain clear chemiluminescence using a conventional smartphone (iPhone 12) camera, ≈0.9 × 10^6^ aubitrary unit (AU) is required (Figure S2e, Supporting Information), but the chemiluminescence resulting from PULSERAA version 1.0 was not sufficient (0.16 × 10^6^ AU).

### 2.6 Application of PULSERAA Version 1.0 to Influenza A Virus Detection

**Figure 4:**
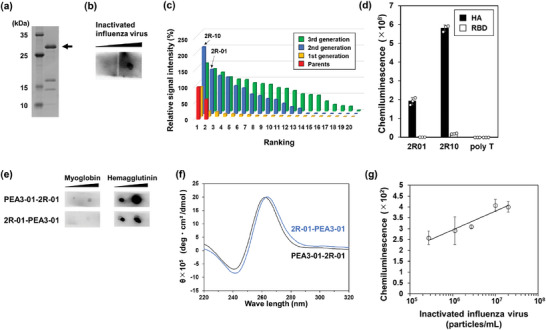
a) SDS‐PAGE analysis after the preparation of anti‐HA scFv‐ST. Lower bands were considered as variable region of heavy chain (12.6 kDa) and ST‐fused variable region of light chain (17.5 kDa), which were degraded at the flexible linker of anti‐HA scFv‐ST during preparation. b) Evaluation of the binding ability of anti‐HA scFv‐ST to the immobilized inactivated influenza A virus (5.4 × 10^7^ and 5.4 × 10^8^ particles) by dot blot assay. c) Summary of in silico maturation based on binding ability of each aptamer candidate. Each bar shows the relative chemiluminescence intensity obtained from the analysis of binding ability using dot blot assays when the chemiluminescence intensity of the parent sequence was defined as 100%, and each sequence was ranked based on the relative chemiluminescence intensity. d) Evaluation of the binding ability of the selected aptamer to HA of influenza A virus and RBD of SARS‐CoV‐2. Data are represented as mean ± S.D. (*n* = 3). e) Evaluation of the binding ability of the bispecific aptamers, PEA3‐01–2R‐01 and 2R‐01–PEA3‐01, against Mb (0.35 and 3.5 µg) and HA (2.0 and 10 pmol) by dot blot assay. f) Circular dichroism spectrum analysis of the bispecific aptamers. The results of PEA3‐01–2R‐01 and 2R‐01–PEA3‐01 are shown in black and blue, respectively. g) Detection of the inactivated influenza A virus. The concentrations of inactivated influenza A virus are represented in logarithmic scale. Data are represented as mean ± S.D. (*n* = 3, R^2^ = 0.958).

The most characteristic feature of PULSERAA version 1.0 is the versatility of the system, as the antibody can be easily replaced by the SC/ST system and the bispecific aptamer can be easily generated via chemical synthesis. Therefore, to confirm the versatility of PULSERAA version 1.0, it was expanded to detecting influenza A virus (A/Taiwan/1/86). Hemagglutinin (HA), a surface protein of the influenza A virus, was selected as the target. Anti‐HA scFv‐ST was designed based on a previous report^[^ ^30^ ^]^ and prepared (**Figure** **4**a). The binding ability of the scFv‐ST to inactivate influenza A virus was also confirmed (Figure 4b). The HA‐binding aptamer, 2R‐01, was then obtained (Figure 4c), and specific binding to HA was confirmed (Figure 4d). Please refer to the Supporting Information for detailed strategies regarding how to obtain aptamer candidates. Next, the bispecific aptamers, PEA3‐01–2R‐01 and 2R‐01–PEA3‐01, were prepared and analyzed for their binding abilities to Mb and HA (Figure 4e). As their structures did not differ (Figure 4f), PEA3‐01–2R‐01, which showed better binding ability, was used in PULSERAA version 1.0. As a result, inactivated influenza A virus was detected within a linear range of 2.8 × 10^5^–2.8 × 10^7^ particles per mL (Figure 4g). Therefore, PULSERAA version 1.0 can be readily adapted to detect multiple types of viruses by substituting only the corresponding antibody and aptamer pair. The use of a combination of antibodies and aptamers as the detection elements would be another advantage. The preparation of different antibody pairs that recognize non‐overlapping surface areas on the same antigen is sometimes difficult. In contrast, aptamers may bind to different sites, such as highly positively charged surfaces and small pockets, which are not amenable for antibody binding. Aptamers can also be obtained more conveniently and strategically using the systematic evolution of ligands by exponential enrichment (SELEX) protocol by precisely regulating competitive selection using a counterpart antibody.

### 2.7 PULSERAA Version 1.1 using Aptamer‐DNAzyme as an Acceptor instead of Mb and Application to Detection of other Virus Variants and Clinical Sample Detection

**Figure 5:**
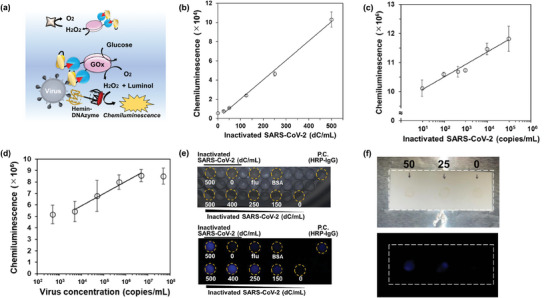
a) A schematic illustration of PULSERAA version 1.1. Hemin‐binding aptamer and hemin are used instead of Mb as the acceptor molecule. b) Detection of inactivated SARS‐CoV‐2 using PULSERAA version 1.1. The data are represented as mean ± S.D. (*n* = 3, R^2^ = 0.998). c) Detection of inactivated SARS‐CoV‐2 Omicron variant using PULSERAA version 1.1. The data are represented as mean ± S.D. (*n* = 2, R^2^ = 0.982). d) Detection of SARS‐CoV‐2 Beta variant from a human throat swab of a COVID‐19 patient. The virus concentration was determined by digital PCR. The data are represented as mean ± S.D. (*n* = 8, R^2^ = 0.985). e) Visualization of inactivated SARS‐CoV‐2‐containing wells using PULSERAA version 1.1. The upper picture was captured under the light source to check the positions of the wells. The lower panel shows a picture obtained using a camera originally installed in an iPhone 12. HRP‐conjugated anti‐mouse IgG was used as the positive control. f) Visualization of inactivated SARS‐CoV‐2 spotted on a nitrocellulose membrane; 50, 25, and 0 dC of the virus were spotted and the places are indicated by arrows. The reaction solution in the dispenser was sprayed and chemiluminescence was imaged using an iPhone 12.

Hemin possesses peroxidase‐mimicking activity at the center of the iron,^[^ ^31^, ^32^ ^]^ and aptamer‐DNAzymes composed of a hemin‐binding aptamer and hemin exert enhanced peroxidase activity.^[^ ^33^ ^]^ A previous report has shown that luminol chemiluminescence obtained from hemin was higher than that obtained from Mb.^[^ ^22^ ^]^ Therefore, to further expand the usability of the PULSERAA system and to increase chemiluminescence intensity for the detection using a camera, we designed PULSERAA version 1.1, integrating aptamer‐DNAzyme as an acceptor instead of Mb (**Figure** **5**a). Here, we used hemin and PS2.M for the aptamer‐DNAzyme and prepared a bispecific aptamer (PS2.M–RBD‐46). CD measurements revealed a negative peak and a positive peak near 240 and 260 nm, respectively, indicating that the bispecific aptamer formed a parallel G4 structure (Figure S3a, Supporting Information).

As a result of inactivated SARS‐CoV‐2 detection, the viral concentration‐dependent increase in chemiluminescence intensity was confirmed in a good linear range of 25–500 dC mL^−1^ (Figure 5b), and the LOD was calculated to be 28 dC mL^−1^, showing that PULSERAA version 1.1 detects the inactivated SARS‐CoV‐2 with high sensitivity. The detection based on enzyme cascades sometimes resulted in poor LOD due to high background signal as a biosensor based on a DNA tweezer based on switchable GOx/DNAzyme cascades has shown.^[^ ^34^ ^]^ In addition, highly sensitive detection in homogeneous immunosensors is also challenging; conventional homogeneous immunosensors based on FRET and AlphaLISA show LODs around sub‐fM at best.^[^ ^35^, ^36^, ^37^, ^38^ ^]^ Nevertheless, the PULSERAA achieved such a highly sensitive detection that the LOD of RBD protein can be calculated as sub‐aM. One reason is sequential enzymatic reactions on the viral surface. Since the number of trimeric spike proteins on a single virus ranges from 20 to 40,^[^ ^39^, ^40^ ^]^ there are ≈120 RBDs on a virus surface, which leads to the accumulation of hydrogen peroxide in a limited space. The accumulation of chemical species in a limited space leads to high sensitivity in biosensors.^[^ ^41^, ^42^ ^]^ Therefore, to support the fact that the accumulation is important for highly sensitive detection by the PULSERAA system, we performed the detection of soluble SARS‐CoV‐2 trimeric spike protein and soluble hemagglutinin, by PULSERAA version 1.1. As shown in Figure S4 (Supporting Information), no signal increase was observed in both cases when the target was not accumulated on the viral surface. Therefore, we clearly demonstrate that the accumulation of the target proteins and sequential enzymatic reactions on the viral surface is responsible for the highly sensitive detection by the homogeneous immunosensor of PULSERAA.

One potential challenge in overcoming a virus pandemic is that amino acid mutations in surface proteins prevent antibodies from binding and allow the virus to evade immune recognition. This also suggests that the accuracy of SARS‐CoV‐2 detection is affected by the decreased affinity of detection antibodies to the variants. Therefore, we used scFv (clone: S309) which can strongly bind to the SARS‐CoV‐2 Omicron variant.^[^ ^43^ ^]^ We prepared the ST‐fused scFv to fabricate the new AEC and applied it to the detection of inactivated SARS‐CoV‐2 Omicron variant (USA/GA‐EHC‐2811C/2021) by PULSERAA version 1.1. As shown in Figure 5c, an inactivated virus concentration‐dependent chemiluminescence increase and a high linear range of 9.5–9.5 × 10^4^ copies per mL was observed. These results strongly demonstrate that PULSERAA is a robust system to be adopted to a variety of virus variants by replacing antibody and/or aptamer pairs.

Next, to investigate the use of PULSERAA version 1.1 in the clinic, we performed the detection of the SARS‐CoV‐2 Beta variant (B.1.351) in a human throat swab of a COVID‐19 patient. As shown in Figure 5d, a virus concentration‐dependent signal increase was observed. A high linear range of 5.0 × 10^3^–5.0 × 10^6^ copies per mL was confirmed. However, the decreased linear range and the large variability were observed when compared to the result of inactivated SARS‐CoV‐2 detection. Viruses including SARS‐CoV‐2 are known to aggregate without appropriate sample treatments such as addition of non‐ionic detergent^[^ ^44^, ^45^ ^]^ or diethylaminoethyl dextran,^[^ ^46^ ^]^ and optimization of pH and ionic strength.^[^ ^47^, ^48^ ^]^ Therefore, virus aggregation may explain the variability of detection by PULSERAA in the clinical sample. Appropriate sample pretreatment may be required for real sample detection with high sensitivity.^[^ ^49^ ^]^ While some optimizations of detection conditions or sample treatments may be required for further use, these results suggest that PULSERAA version 1.1 can work even in the presence of biological contaminants.

### 2.8 Visualization of the Inactivated SARS‐CoV‐2‐Containing Spots using PULSERAA Version 1.1

Finally, we focused on the visualization of virus‐containing spots by PULSERAA version 1.1 because it achieved homogeneous virus detection. First, we investigated the mixing ratio of hemin with the bispecific aptamer. As a result, the chemiluminescence intensity increased with an increase in mixing ratio (Figure S3b, Supporting Information). The use of a mixing ratio of 1:1 resulted in chemiluminescence below the iPhone 12 camera detection threshold, and therefore it was necessary to add hemin in excess at a concentration of 50 µm. Under the optimized conditions, inactivated SARS‐CoV‐2 was visually detected in a virus concentration‐dependent manner using PULSERAA version 1.1 with smartphone camera imaging (Figure 5e). Then, to enable practical visualization of virus‐containing spots using PULSERAA version 1.1, we used a dispenser to conveniently spray the detection reagent. While the chemiluminescence intensity was considerably lower than that observed with plate imaging, the spot of inactivated SARS‐CoV‐2 immobilized on a nitrocellulose membrane was successfully visualized by smartphone camera imaging with the immobilization amount‐dependent manner (Figure 5f).

To date, a number of SARS‐CoV‐2 detection methods including the proximity‐based luminescence and homogeneous method^[^ ^50^ ^]^ have been proposed (Table S2, Supporting Information). RT‐qPCR is the gold standard for SARS‐CoV‐2 detection, but it is time‐consuming (<4 h).^[^ ^51^ ^]^ Screening based on lateral flow immunoassay requires just 15–20 min,^[^ ^52^ ^]^ but it sometimes lacks sensitivity. Furthermore, none of these methods can achieve the visualization of virus‐containing spots. PULSERAA showed an equivalent LOD to RT‐qPCR within a short time (≈15 min) and achieved the visualization of virus‐containing spots by spraying the detection reagent through a conventional smartphone camera. Our result is the first report to show the visualization of infectious spots, which contributes to the prevention of infections during future pandemics. In the visualization assay, we spotted the inactivated SARS‐CoV‐2 on the membrane and the sample was allowed to dry. Then, an aliquot of the sample solution containing the AEC, hemin, and the bispecific aptamer was dropped onto the membrane, and after incubation for 20 min, the detection solution was sprayed onto the membrane. The resulting chemiluminescence (Figure 5f) shows that the detection reagents are stable after being exposed to the air at room temperature for over 20 min, suggesting that detection by PULSERAA is also compatible with an outdoor environment. Incorporation of a two‐solution (solution A containing the AEC, bispecific aptamer, and hemin, and solution B containing glucose, luminol, and catalase) sequential spraying system, is now being pursued to improve PULSERAA further for rapid detection and prevention of infectious diseases in the near future.

### 2.9 PULSERAA Version 2.0 using HRP‐Conjugated Antibody as the Acceptor

To further broaden the accessibility to the PULSERAA system and to increase the output chemiluminescence signal, we also designed PULSERAA version 2.0, consisting of the AEC and a commercially available HRP‐conjugated antibody (HRP‐Ab) (Figure S5a, Supporting Information). As shown in Figure S5b (Supporting Information), the viral concentration‐dependent increase in chemiluminescence intensity was confirmed with a linear range of 25–250 dC mL^−1^. Because 25 dC mL^−1^ was the lowest concentration with a reproducible signal in our assay, this value was used as the LOD. These results indicate that PULSERAA 2.0 is versatile and accessible because of the utilization of commercially available HRP‐Ab.

## 3 Conclusion 

In this study, a convenient and universal viral detection and visualization platform, PULSERAA, was designed, and homogeneous detection and visualization of virus‐containing spots were achieved using sequential enzymatic reactions on the viral surface. PULSERAA enabled successful detection of inactivated SARS‐CoV‐2 variants and inactivated influenza A virus with high sensitivity. Furthermore, PULSERAA enabled the detection of infectious SARS‐CoV‐2 in a throat swab due to the accumulation of antigenic targets on the viral surface and local sequential enzymatic reactions. These results indicate that PULSERAA can be readily adapted to detect a wide range of harmful viruses and that the three versions of PULSERAA systems can be used in different situations as needed in the future. Finally, to the best of our knowledge, PULSERAA is the first reported system for on‐site visualization of virus‐containing spots. Therefore, PULSERAA can enable infection control in public spaces through the visualization of infectious spots containing lethal viruses on doorknobs or elevator buttons.

## 4 Experimental Section

### Construction of Expression Vectors

To construct an expression vector for anti‐RBD scFv, the amino acid sequence was designed based on the parental Fab format sequence (clone: CB6 and clone: S309) reported by Shi et al.^[^ ^19^ ^]^ and Pinto et al.^[^ ^43^ ^]^ Anti‐HA scFv was previously reported by Li et al.^[^ ^30^ ^]^ Both codon‐optimized genes were synthesized and inserted into T7 promoter‐based vectors.^[^ ^53^ ^]^ ST was genetically fused to the C‐termini of both scFvs. The SC gene was fused to either the N‐ or C‐terminus of the GOx derived from *A. niger*.

### Production of Recombinant Antibodies

Both anti‐RBD scFv‐ST and anti‐HA scFv‐ST (clone: 3JA18) were prepared using *Escherichia coli* BL21 (DE3). For production of anti‐RBD scFv‐ST (clone: CB6), it was prepared from the insoluble fraction and refolding process. The cultivation conditions were the same as described previously.^[^ ^16^ ^]^ Wet cells were obtained after centrifugation at 4800 × *g* for 20 min and resuspended in 20 mm phosphate buffer (pH 8.0). The cells were disrupted via ultrasonication and the intracellular insoluble fraction was obtained after centrifugation at 15000 × *g* for 20 min. The insoluble fraction of scFv was prepared using previously reported refolding protocols, with some modifications.^[^ ^54^, ^55^ ^]^ Briefly, the insoluble fraction was solubilized in 20 mm phosphate buffer containing 6 m guanidium hydrochloride (Nippon Carbide Industries Co. Inc., Tokyo, Japan) (pH 8.0). After centrifugation at 15000 × *g* for 30 min, the solubilized fraction was purified using Ni Sepharose 6 Fast Flow (Cytiva, Tokyo, Japan). To remove guanidine, the purified fraction was dialyzed twice against 20 mm phosphate buffer containing 0.5 m L‐arginine (Nacalai Tesque, Kyoto, Japan) (pH 8.0) at 4 °C. The mixture was then incubated twice with 20 mm phosphate buffer (pH 6.5) at 4 °C. The resulting refolded sample was further purified using Superdex 200 Increase 10/300 GL (Cytiva) in 20 mm phosphate buffer (pH 6.5) containing 200 mm NaCl.

Anti‐RBD scFv‐ST (clone: S309) and anti‐HA scFv were prepared using *E. coli* BL21 (DE3) and purified from the culture supernatant following a previously described protocol.^[^ ^16^ ^]^

### Production of Recombinant SC Fused GOx

SC‐fused GOxs were prepared using *E. coli* BL21(DE3). The transformants were cultured in 100 mL Luria–Bertani (LB) medium at 37 °C. When the OD reached 0.6, isopropyl‐β‐d‐thiogalactopyranoside (IPTG) was added (0.5 mm) to induce protein expression, and the cells were cultured at 37 °C and 150 rpm for 24 h. The cells were collected via centrifugation at 4800 × *g* at 4 °C for 20 min and resuspended in 20 mm phosphate buffer (pH 7.0). Recombinant GOx was prepared using refolding processes as reported previously.^[^ ^56^ ^]^ Briefly, the cells were disrupted via ultrasonication and the insoluble fraction was obtained after centrifugation at 10 000 × *g* at 4 °C for 20 min. The insoluble pellet was washed with 20 mm Tris‐HCl containing 100 mm NaCl, 1 mm ethylenediaminetetraacetic acid (EDTA), and 1% Triton‐X (pH 8.0) at 1500 rpm and 4 °C for 30 min, and the pellet was collected after centrifugation at 10 000 × *g* and 4 °C for 10 min. This process was repeated twice. Triton‐X was washed with 20 mm Tris‐HCl containing 100 mm NaCl and 1 mm EDTA (pH 8.0) at 1500 rpm and 4 °C for 1 h, and the pellet was collected after centrifugation at 10 000 × *g* at 4 °C for 10 min. Next, the pellet was washed with 20 mm Tris‐HCl containing 2 m urea (pH 8.0) at 1500 rpm for 30 min at 4 °C, and collected after centrifugation at 10 000 × *g* at 4 °C for 10 min. Finally, the pellet was solubilized in 20 mm Tris‐HCl containing 8 m urea and 30 mm dithiothreitol (pH 8.0) at 1500 rpm and 4 °C for >4 h. The solubilized supernatant was collected after centrifugation at 10 000 × *g* at 4°C for 10 min and diluted to 0.05 mg mL^−1^ total protein concentration using 20 mm phosphate buffer containing 1 mm oxidized glutathione, 1 mm reduced glutathione, 0.05 mm flavin adenine dinucleotide, and 10% (w/v) glycerol (pH 7.5) and incubated at 10 °C for >90 h. Next, the samples were concentrated using ultrafiltration with Amicon Ultra‐15 30 K (30 kDa cutoff, Merck Millipore, Burlington, MA, USA) and dialyzed against 20 mm sodium acetate buffer (pH 5.0) at 4 °C for 12 h, followed by that against 20 mm phosphate buffer (pH 7.0) overnight at 4 °C. Finally, some aggregates were removed via centrifugation at 15 000 × *g* and 4 °C for 10 min.

### ELISA

SARS‐CoV‐2 spike RBD recombinant protein (Sino Biological, Inc., Beijing, China) was immobilized (200 nM) onto a 96‐well MaxiSorp™ plate using the carbonate‐bicarbonate buffer (Thermo Fisher Scientific, Waltham, MA, USA). After washing three times with Tris‐buffered saline containing 0.05% (v/v) Tween 20 (TBS‐T), the plate was blocked with TBS‐T containing 1% (w/v) BSA at 25 °C for 1 h. After washing, various concentrations (0–200 nM) of anti‐RBD scFv‐ST were added and incubated at 25 °C for 1 h. After washing three times, HRP‐conjugated anti‐(His)_6_ tag IgG was added and incubated at 25 °C for 1 h. Finally, after three washes, ELISA POD substrate TMB solution (Nacalai Tesque) was added, and the plate was incubated at 25 °C for several minutes. The reaction was stopped and the absorbance at 450 nm was measured using a plate reader (Multiskan™ GO, Thermo Fisher Scientific).

### Fabrication of AECs

To prepare the AECs, 10 µm ST‐fused scFv was mixed with 5 µm SC‐GOx and incubated overnight at 4 °C. The formation of AEC was confirmed using SDS‐PAGE under reducing conditions after adding a loading buffer containing 125 mm Tris‐HCl, 4% sodium dodecyl sulfate, 20% glycerol, 3.1% dithiothreitol, and 0.01% bromophenol blue (pH 6.8), and boiling at 95 °C for 10 min.

### Analysis of Enzymatic Activity

Enzymatic activities of SC‐fused GOxes and the AEC were measured by monitoring the formation of hydrogen peroxide using 1.5 mm 4‐aminoantipyrine (4AA) (Fujifilm Wako Pure Chemical Corporation, Osaka, Japan), 1.5 mm N‐ethyl‐N‐(2‐hydroxy‐3‐sulfopropyl)‐3‐methylaniline (TOOS) (Dojindo Laboratories, Kumamoto, Japan), and 2 U HRP (Sigma‐Aldrich, St. Louis, MO, USA) in 20 mm phosphate buffer (pH 7.0). The quinoneimine dye was generated under these conditions, and enzymatic activity was calculated from the absorbance at 555 nm (Figure S1b, Supporting Information). Here, one unit of specific activity was defined as the amount of enzyme that produces 1 µmol of the quinoneimine dye in a min at 25 °C. The kinetic parameters, *K*_M_ and *V*_max_, were calculated by the Hanes–Woolf plot.

### Biolayer Interferometry Analysis

The SARS‐CoV‐2 spike RBD recombinant protein was biotinylated using EZ‐Link™ NHS‐PEG4‐Biotin No‐Weigh™ Format (Thermo Fisher Scientific). It was then captured to an Octet® streptavidin‐immobilized sensor tip (Sartorius, Goettingen, Germany) for 150 s. Anti‐RBD scFv‐ST and the AEC were diluted using a running buffer (20 mm phosphate buffer (pH 7.0) containing 0.005% Tween‐20) to 12–500 nm scFv‐ST and 0.52–500 nm AEC, and incubated with the modified sensor tip. The association time was set to 120 s. Subsequently, the tip was immersed in the running buffer and allowed to dissociate. The dissociation time was set at 120 s. Each sensorgram was generated using the binding signal and set to zero when the association step started. Appropriate corrections were performed because of the signal drifts between the association and dissociation steps. The gaps caused by the corrections are shown in the figure. The kinetic parameters were obtained by applying 1:1 Langmuir global fitting.

### Aptamer Selection

RBD‐binding aptamers were designed using CoV2‐RBD‐4C, suggesting the formation of a hairpin structure as a template.^[^ ^23^ ^]^ The G4 structure was considered suitable for binding to the RBD because it harbored a positively charged area. CoV‐2‐RBD‐4C has a G‐rich sequence predicted to form a G4 structure at the 3’‐end. It has also been reported that the 3’‐end of CoV2‐RBD‐4C was a key region for RBD binding with a network of hydrogen bonds. Therefore, only the 3’‐end sequence of CoV2‐RBD‐4C was used as a SARS‐CoV‐2 binding aptamer and renamed it RBD‐46.

Aptamer candidates against HA, a surface protein of influenza virus, were obtained using the in silico maturation (ISM) method.^[^ ^57^ ^]^ First, two aptamers, RHA0385^[^ ^58^ ^]^ and ApII,^[^ ^59^ ^]^ were used as templates, and primary sequences were designed by shuffling the loop region of the G4 structure of the template sequences and introducing a random single‐nucleotide mutation. After three rounds of ISM, all 60 candidates and their parent sequences were subjected to analysis of their binding abilities to HA using enzyme‐linked oligonucleotide assay (ELONA). ELONA was performed as described below. HA (Sino Biological, Inc.) was immobilized onto a 96‐well MaxiSorp™ plate using the carbonate‐bicarbonate buffer (Thermo Fisher Scientific) at 4 °C overnight. After washing three times with TBS‐T, the plate was blocked with TBS‐T containing 2% (w/v) BSA at 25 °C for 1 h under gentle shaking (700 rpm). After washing, each aptamer candidate (500 nm) was added and incubated at 25 °C for 1 h under gentle shaking (700 rpm). After washing three times, 5000‐fold diluted HRP‐conjugated NeutrAvidin (Thermo Fisher Scientific) was added and incubated at 25 °C for 1 h under gentle shaking (700 rpm). Finally, after three washes, BM Chemiluminescence ELISA Substrate (Roche, Basel, Switzerland) was added, and the plate was incubated at 25 °C for 5 min. Chemiluminescence was measured using a plate reader (Varioskan Flash, Thermo Fisher Scientific). Then, the candidate showed high binding to HA was selected as the HA‐binding aptamer.

All aptamer sequences are summarized in Table S1 (Supporting Information).

### Dot Blot Assay

All DNA oligonucleotides were obtained from Eurofin Genomics (Tokyo, Japan). They were then mixed with 3’‐biotinylated oligonucleotides for binding analysis, incubated at 95 °C for 10 min, and gradually cooled to 25 °C for 2 h.

Mb (Life Diagnostics, West Chester, PA, USA) and inactivated SARS‐CoV‐2 virus (Qnostics, Scotland, UK) were spotted and immobilized on a nitrocellulose membrane. The membrane was incubated with 10 mm sodium phosphate buffer (pH 7.0) containing 10 mm KCl, 0.05% (v/v) Tween‐20, and 2% (w/v) BSA for 1 h to prevent non‐specific binding. After washing with 10 mm sodium phosphate buffer (pH 7.0) containing 10 mm KCl and 0.05% (v/v) Tween‐20, the membranes were incubated with 100 nm oligonucleotides at 25 °C for 1 h. After washing, the membranes were incubated with streptavidin alkaline phosphatase (Promega, Madison, WI, USA) 1000‐fold diluted in the same buffer at 25 °C for 1 h. Following the washing steps, chemiluminescence was detected using LAS 4000 mini (Cytiva) and ready‐to‐use CDP‐Star (Thermo Fisher Scientific).

### Circular Dichroism Spectroscopy

Circular dichroism spectroscopy was performed using J‐820 (JASCO Co., Ltd., Tokyo, Japan). The scan range was 320–220 nm and the scan rate was 100 nm min^−1^. Measurements were repeated five times for each aptamer at 25 °C. The corresponding background spectrum of each buffer or protein was subtracted, and smoothing treatment was performed according to the manufacturer's protocol.

### Homogeneous Virus Detection using PULSERAA

In PULSERAA version 1.0, 10 µm of the bispecific aptamer (PEA3‐01–RBD‐46), 2 µm of Mb, and 1 µm of the AEC were prepared by diluting with 10 mm sodium phosphate buffer containing 10 mm KCl (pH 7.0). Each 5 µL of the component was mixed equally in a 96‐well protein low‐binding LBS OptiPlate‐96 (Perkin Elmer, Inc., Waltham, MA, USA). Then, 5 µL of inactivated virus sample was spiked into the mixed solution and incubated at 25 °C. According to the manufacturer's instructions, inactivated SARS‐CoV‐2 Wuhan strain was inactivated by heat treatment and gamma irradiation, and inactivated SARS‐CoV‐2 Omicron strain was inactivated by heat treatment at 65 °C for 30 min. The plate was sealed to prevent the solution from drying during the incubation. Finally, 80 µL of Solution A of BM chemiluminescence ELISA substrates (POD) (Roche, Basel, Switzerland) supplemented with 250 mm glucose and 30 ng mL^−1^ catalase (Fujifilm Wako Pure Chemical Corporation) was added.

In PULSERAA version 1.1, 10 µm of bispecific aptamer (PS2.M–RBD‐46), 50 µm of hemin (Tokyo Chemical Industry Co., Ltd., Tokyo, Japan), and 1 µm of the AEC were prepared, and each 5 µL of the component was mixed in a 96‐well protein low‐binding LBS OptiPlate‐96 (Perkin Elmer, Inc.) and then incubated with the inactivated SARS‐CoV‐2 or SARS‐CoV‐2 Beta variant (B.1.351) in PBS‐diluted throat swab from a COVID‐19 patient under the same conditions as for PULSERAA version 1.0. The SARS‐CoV‐2 concentration in the throat swab was determined using a digital PCR kit (qScript XLT 1‐Step RT‐qPCR ToughMix; Quantabio, Cummings Center, MA, USA) following the manufacturer's instruction. Solution A of BM chemiluminescence ELISA substrates (POD) supplemented with 250 mm glucose and 30 ng mL^−1^ catalase was used as the reaction solution, and chemiluminescence was immediately measured. The investigation using infectious SARS‐CoV‐2 was undertaken at the Tokai University Medical School Biosafety Level 2 (BSL2) laboratory. For detection after spraying the reaction solution consisting of solution A supplemented with 250 mm glucose and 30 ng mL^−1^ catalase, 50, 25, or 0 dC of inactivated SARS‐CoV‐2 was spotted onto a nitrocellulose membrane and dried for a few minutes. Then, 15 µL of a solution containing 333 nm AEC, 3.3 µm bispecific aptamer, and 16.7 µm hemin was dropped onto each spot and incubated for 20 min at 25 °C. Finally, 800 µL of the reaction solution was placed in a dispenser and sprayed over the membrane, followed by imaging with an iPhone 12 camera.

In PULSERAA version 2.0, commercially available HRP‐anti‐SARS‐CoV‐2 IgG (Abcam, Cambridge, UK) was diluted using 10 mm sodium phosphate buffer containing 10 mm KCl (pH 7.0). It was equally mixed with 1 µm of the AEC and various concentrations of inactivated SARS‐CoV‐2 and incubated at 25 °C for an hour in an LBS OptiPlate‐96. Solution A of BM chemiluminescence ELISA substrates (POD) supplemented with 250 mm glucose and 30 ng mL^−1^ catalase was added, and chemiluminescence was immediately measured using Varioskan Flash (Thermo Fisher Scientific).

### Statistical Analysis

Data were presented as the mean ± standard deviation (SD) unless otherwise stated. Statistical analysis for each experiment was described in the figure legends. The numbers of technical and biological replicates for each result were described in figure legends. All the statistical analyses were performed using MATLAB R2024a (Mathworks, Natick, MA, USA). The significance of the results was analyzed by Student's *t*‐test (two‐tailed), or one‐way ANOVA, and followed by Tukey's post hoc test. *p* < 0.05 was considered to be a statistically significant difference. Each *p*‐value was indicated in the results.

## Conflict of Interest

The authors declare no conflict of interest.

## Ethics Approval Statement and Patient Consent Statement

The study using a biological sample obtained from a patient was approved by the Review Board of Tokai University (approval number: 20R‐052) and National Hospital Organization Omuta National Hospital (approval number: 22R‐050). The patient provided verbal as well as written informed consent to participate in this study.

## Supporting information



Supporting Information are available from the link below, and other references are included.

## Data Availability

The data that support the findings of this study are available on request from the corresponding author. The data are not publicly available due to privacy or ethical restrictions.
