# Supplementary material for: Proximity‐Unlocked Luminescence by Sequential Enzymatic Reactions from Antibody and Antibody/Aptamer (PULSERAA): A Platform for Detection and Visualization of Virus‐Containing Spots
Source: Adv Sci (Weinh). 2024 Sep 24;11(43):2403871. doi: 10.1002/advs.202403871 (PMC11578312; doi:10.1002/advs.202403871)
Supplement: Supplementary file 1 — Supporting Information are available from the link below, and other references are included. [file ADVS-11-2403871-s001.pdf]

ADVANCED SCIENCE
Open Access

Supporting Information

for *Adv. Sci.*, DOI 10.1002/advs.202403871

Proximity-Unlocked Luminescence by Sequential Enzymatic Reactions from Antibody and Antibody/Aptamer (PULSERAA): A Platform for Detection and Visualization of Virus-Containing Spots

Daimei Miura, Wakana Hayashi, Kensuke Hirano, Ikkei Sasaki, Kaori Tsukakoshi, Hidehumi Kakizoe, Satomi Asai, Christopher J. Vavricka, Hitoshi Takemae, Tetsuya Mizutani, Wakako Tsugawa, Koji Sode, Kazunori Ikebukuro and Ryutaro Asano**

Supporting information

Proximity-Unlocked Luminescence by Sequential Enzymatic Reactions from Antibody and Antibody/Aptamer (PULSERAA): A Platform for Detection and Visualization of Virus-Containing Spot

Daimei Miura, Wakana Hayashi, Kensuke Hirano, Ikkei Sasaki, Kaori Tsukakoshi, Hidehumi Kakizoe, Satomi Asai, Christopher J. Vavricka, Hitoshi Takemae, Tetsuya Mizutani, Wakako Tsugawa, Koji Sode, Kazunori Ikebukuro*, and Ryutaro Asano*

*Corresponding Authors

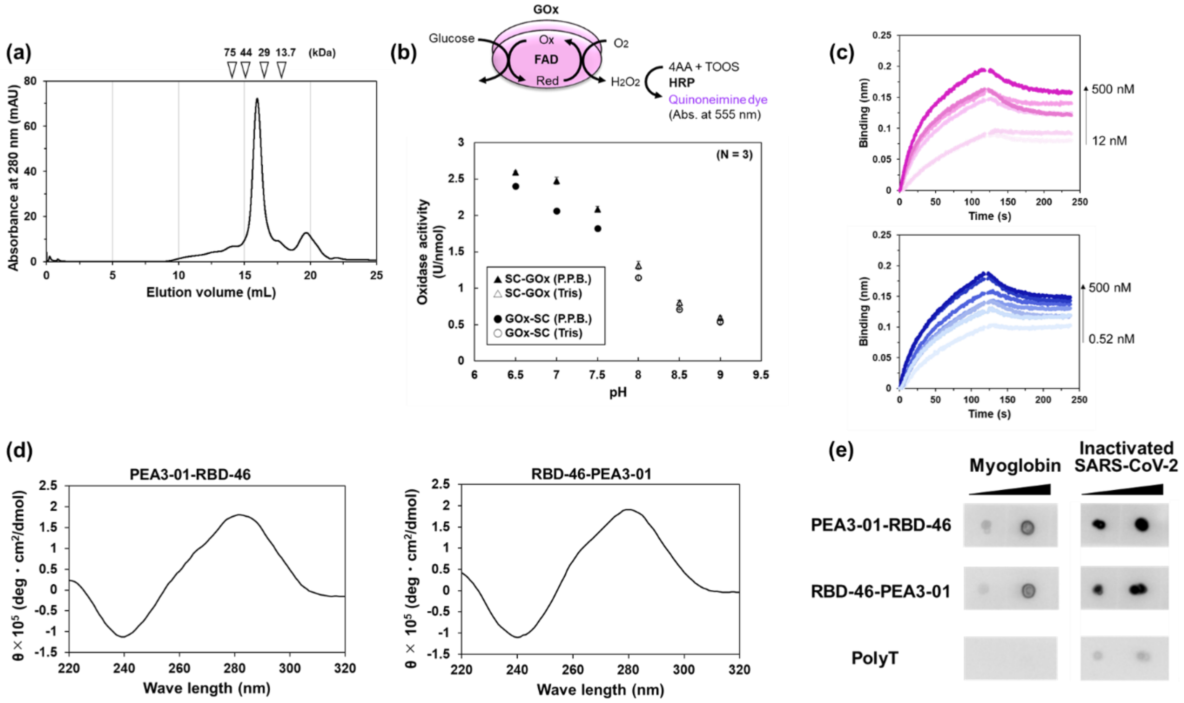

Figure S1 Preparation and characterization of the components for PULSERAA version 1.0. (a) Gel filtration chromatogram for preparation of anti-RBD scFv-ST. (b) A schematic illustration of GOx activity assay and the result of pH scouting of SC-fused GOx. For pH 6.5–7.5, 20 mM potassium phosphate buffer was used and is represented by filled markers. For pH 8–9, 20 mM Tris-HCl buffer was used and is represented by empty markers.

(c) Sensorgrams obtained using biolayer interferometry for antibody-binding analysis of anti-SARS-CoV-2 scFv-ST (top figure) the AEC (bottom figure). Relatively large signal drifts were observed between the association and dissociation steps. In these figures, the gap was subtracted and the plot caused by the drift was removed, which is shown as a blank in the plot. Fitting was performed following the manufacturer's instructions using raw data, and the Chi^2 values are shown in Table 2. (d) Circular dichroism spectrum analysis of the bivalent aptamers folded in 10 mM potassium acetate (pH 5.0). (e) Evaluation of the binding ability of the bispecific aptamers, PEA3-01–RBD-46 and RBD-46–PEA3-01, folded in 10 mM potassium acetate buffer (pH 5.0) against Mb and inactivated SARS-CoV-2 using aptamer blotting. PolyT was used as the negative control.

Receptor binding domain, RBD; single-chain variable fragment, scFv; SpyTag, ST; SpyCatcher, SC; glucose oxidase, GOx; 4-aminoantipyrine, 4AA; N-ethyl-N-(2-hydroxy-3-sulfopropyl)-3-methylaniline, TOOS, antibody-enzyme complex, AEC

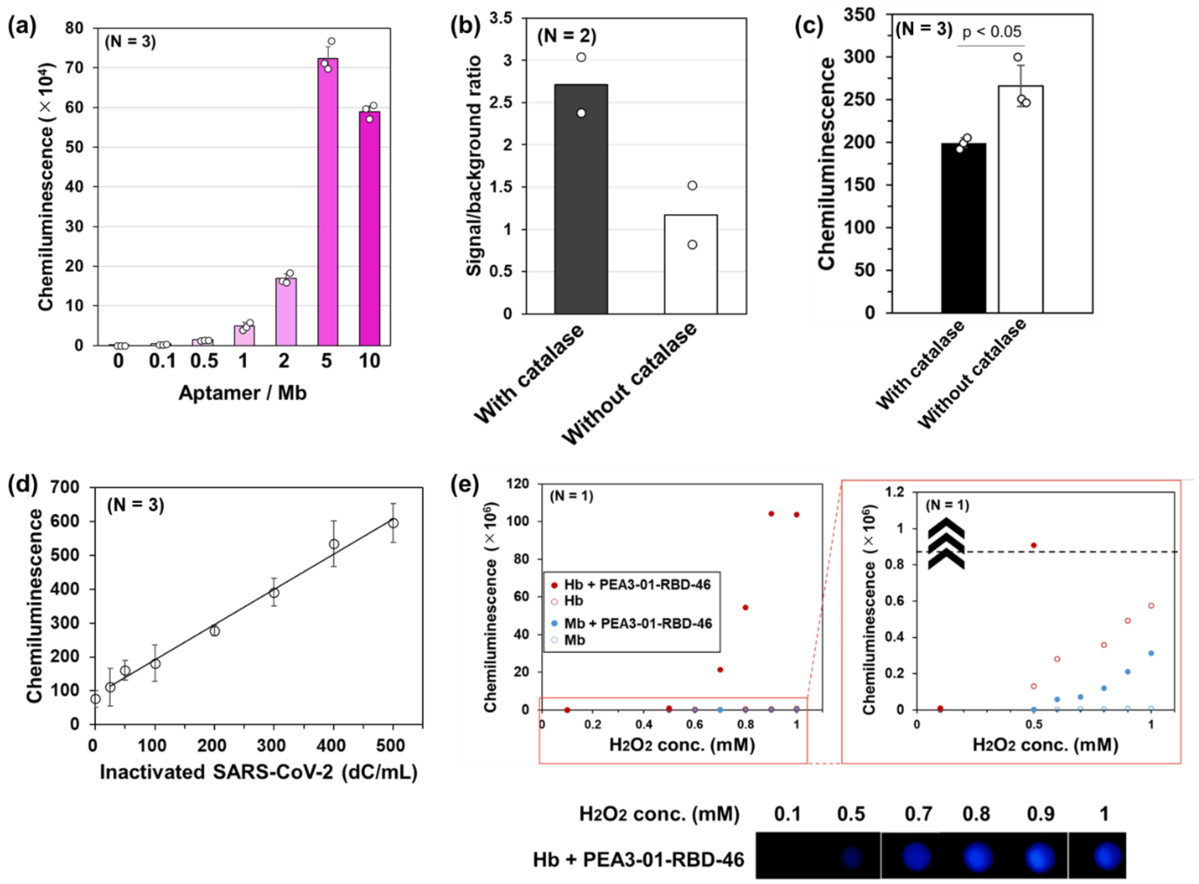

Figure S2 Optimization and investigation of PULSERAA version 1.0. (a) Optimization of the suitable mixing ratio of the bispecific aptamer (PEA3-01–RBD-46) and Mb. (b) The effect of addition of catalase to the reaction solution by the comparison of signal/background ratio calculated from the detection of inactivated SARS-CoV-2 (300 dC/mL) by PULSERAA version 1.0. (c) Chemiluminescence background signals in the presence and the absence of catalase. The detection scheme was the same as described in the manuscript. Statistical analysis was performed using the Student's t-test. Data are represented as mean ± S.D. (n = 3). (d) Inactivated SARS-CoV-2 detection using a handmade detection solution composed of Tris-HCl (pH 8.0) supplemented with 100 μM luminol and 200 mM glucose. Each concentration was described as the final concentration. Data are represented as mean ± S.D. (n = 3). (e) Investigation of the threshold to detect the chemiluminescence using an iPhone 12.

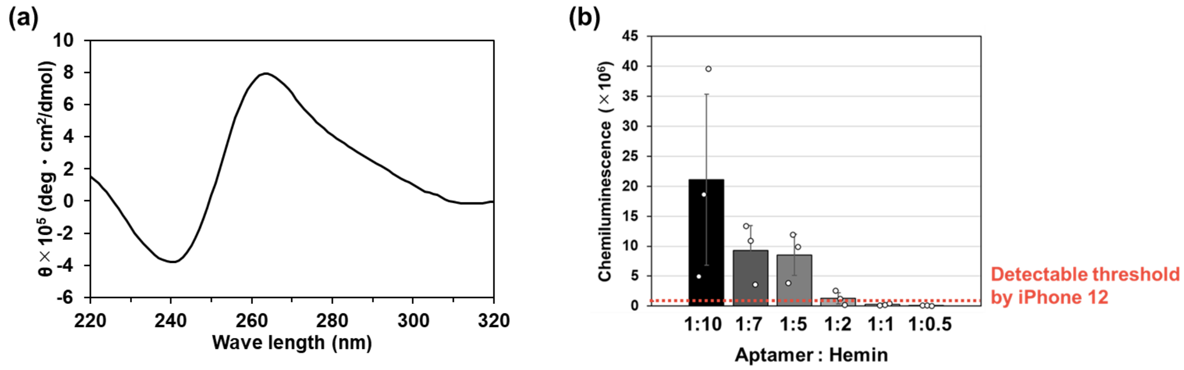

Figure S3 Optimization of detection and visualization conditions for PULSERAA version 1.1. (a) Circular dichroism spectrum analysis of the bivalent aptamer, PS2.M–RBD-46 folded in 10 mM sodium phosphate containing 10 mM potassium chloride (pH 7.0).

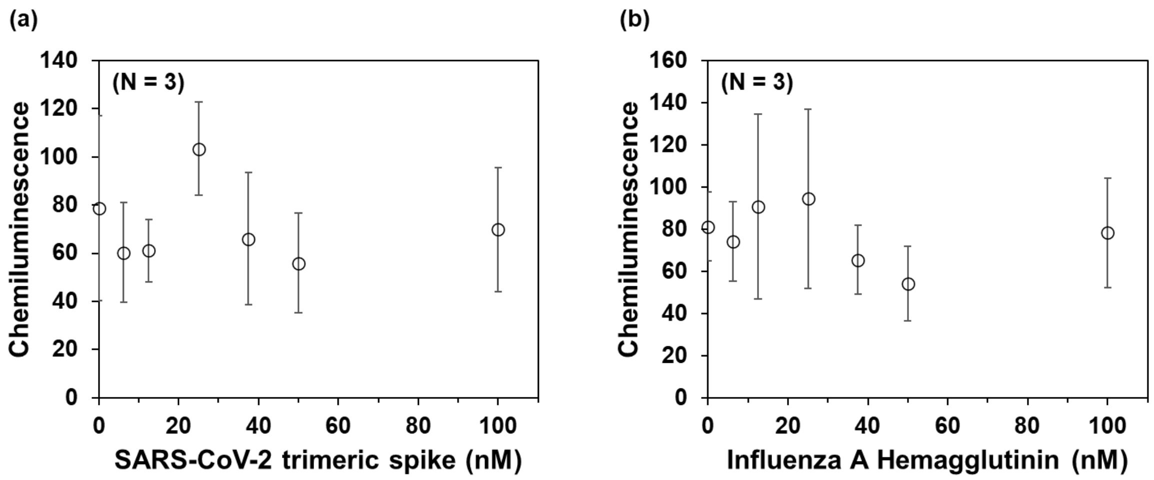

Figure S4 Detection of (a) SARS-CoV-2 trimeric spike protein, (b) influenza A hemagglutinin protein by PULSERAA version 1.1. Here, each concentration of proteins was mixed with the corresponding AEC, the corresponding bispecific aptamer, and hemin instead of each inactivated virus. Other detection procedures were the same as the detection by PULSERAA version 1.1 described in the manuscript. All the data represented as mean ± S.D. (n = 3).

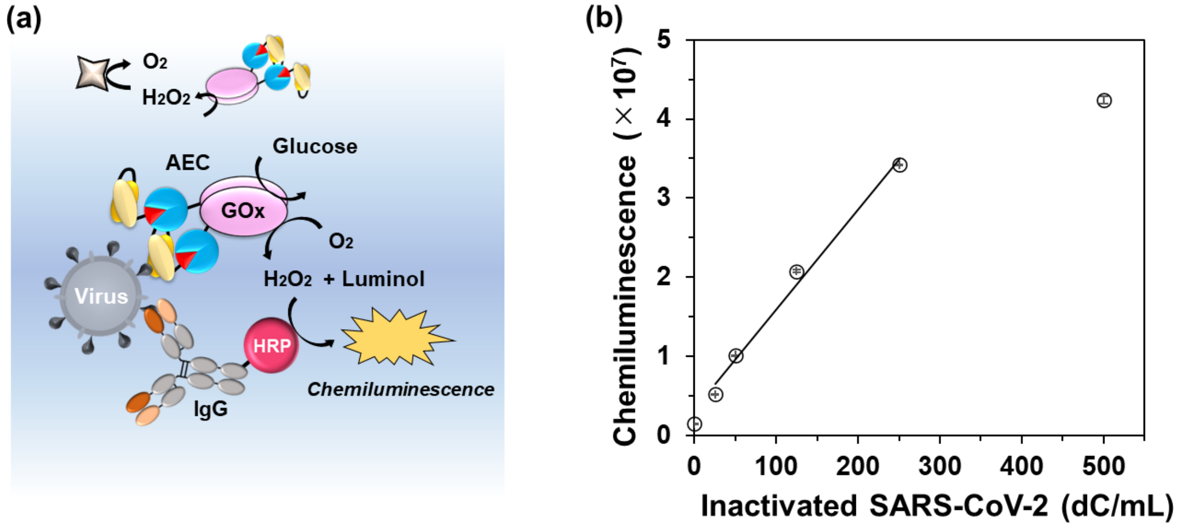

Figure S5 Inactivated SARS-CoV-2 detection by PULSERAA version 2.0. (a) A schematic illustration of PULSERAA version 2.0. (b) A calibration curve of inactivated SARS-CoV-2 detection by PULSERAA version 2.0. Data represent as mean ± S.D. (n = 3, R^2 = 0.989).

Table S1
Aptamers used in this work

Name	Sequence (5′-3′)	Length (mer)
PEA3-01	GGGCGGGTTGGGCTGGG	17
RBD-46	GGGGCTGCTCGGGATTGCGGATATGG	26
PEA3-01–RBD-46	GGGCGGGTTGGGCTGGGtttGGGGCTGCTC GGGATTGCGGATATGG	46
RBD-46–PEA3-01	GGGGCTGCTCGGGATTGCGGATATGGtttGG GCGGGTTGGGCTGGG	46
PS2.M–RBD-46	GTGGGTAGGGCGGGTTGGtttGGGGCTGCT CGGGATTGCGGATATGG	47
PolyT	TTTTTTTTTTTTTTTTTTTTTTTTTTTTTTTT	32
2R-01	TTGGGGTTATTTTGGGTGTGGTGGGTGGG GGTT	33
PEA3-01–2R-01	GGGCGGGTTGGGCTGGGtttTTGGGGTTATT TTGGGTGTGGTGGGTGGGGGTT	53
2R-01–PEA3-01	TTGGGGTTATTTTGGGTGTGGTGGGTGGG GGTTtttGGGCGGGTTGGGCTGGG	53
2R-10	TTGGGGTTATTTTGGGAGGGTGTTGTGGG GGTG	33

Table S2

Comparison of analytical performances with other potential methods

	LOD (copies/mL)	Detection time (min)	On-site visualization of virus-containing spot	References
RT-qPCR	50	< 240	No	[60]
Immunochromatography	5.0×10^5	20-30	No	[61]
Commercial Immunochromatography (Core Test, China)	3.1×10^3	10	No	[62]
Commercial Immunochromatography (ESPLINE, Japan)	2.8×10^5	30	No	[62]
Microfluidic fluorescence immunoassay (LumiraDx)	4.3×10^4	15	No	[62]
Proximity-based bioluminescent sensor	-	60	No	[50]
Microcantilever-based sensor	100	5	No	[63]
Differential pulse voltammetry	200	> 180	No	[64]
Electrochemical impedance spectroscopy	38.6	30	No	[65]
FRET	1.4×10^{11}	25	No	[66]
PULSERAA	28	15-60	Yes	This work

References

[50] Y. Ni, B. J. H. M. Rosier, E. A. van Aalen, E. T. L. Hanckmann, L. Biewenga, A. M. M. Pistikou, B. Timmermans, C. Vu, S. Roos, R. Arts, W. Li, T. F. A. de Greef, M. M. G. J. van Borren, F. J. M. van Kuppeveld, B. J. Bosch, M. Merkx, Nat. Commun., 2021, 12, 1–12.

[60] X. Hu, L. Zhu, Y. Luo, Q. Zhao, C. Tan, X. Chen, H. Zhang, X. Hu, L. Lu, Y. Xiao, S. Huang, Y. He, J. X. L. Sim, S. Su, F. Wang, Y. Peng, J. Wang, Y. Guo, T. Zhong, Clin. Chim. Acta, 2020, 511, 143–148.

[61] Y. Yamaoka, K. Miyakawa, S. S. Jeremiah, R. Funabashi, K. Okudela, S. Kikuchi, J. Katada, A. Wada, T. Takei, M. Nishi, K. Shimizu, H. Ozawa, S. Usuku, C. Kawakami, N. Tanaka, T. Morita, H. Hayashi, H. Mitsui, K. Suzuki, D. Aizawa, Y. Yoshimura, T. Miyazaki, E. Yamazaki, T. Suzuki, H. Kimura, H. Shimizu, N. Okabe, H. Hasegawa, A. Ryo, Cell Reports Med., 2021, 2, 100311.

[62] K. Kontogianni, D. Bengey, D. Wooding, K. Buist, C. Greenland-Bews, C. T. Williams, M. de Vos, V. S. Santos, C. Escadafal, E. R. Adams, T. Edwards, A. I. Cubas-Atienzar, J. Brazilian Soc. Trop. Med., 2022, 55, e0016-2022.

[63] D. K. Agarwal, V. Nandwana, S. E. Henrich, V. P. V. N. Josyula, C. S. Thaxton, C. Qi, L. M. Simons, J. F. Hultquist, E. A. Ozer, G. S. Shekhawat, V. P. Dravid, Biosens. Bioelectron., 2022, 195, 113647.

[64] H. Zhao, F. Liu, W. Xie, T. C. Zhou, J. OuYang, L. Jin, H. Li, C. Y. Zhao, L. Zhang, J. Wei, Y. P. Zhang, C. P. Li, Sensors Actuators, B Chem., 2021, 327, 128899.

[65] V. J. Vezza, A. Butterworth, P. Lasserre, E. O. Blair, A. MacDonald, S. Hannah, C. Rinaldi, P. A. Hoskisson, A. C. Ward, A. Longmuir, S. Setford, E. C. W. Farmer, M. E. Murphy, D. K. Corrigan, Chem. Commun., 2021, 57, 3704–3707.

[66] T. Zhao, W. Yan, F. Dong, X. Hu, Y. Xu, Z. Wang, Y. Shen, W. Wang, Y. Zhao, W. Wei, Microchim. Acta, 2022, 189, 268.
